# Ten considerations for open peer review

**DOI:** 10.12688/f1000research.15334.1

**Published:** 2018-06-29

**Authors:** Birgit Schmidt, Tony Ross-Hellauer, Xenia van Edig, Elizabeth C Moylan

**Affiliations:** 1 State and University Library, University of Göttingen, Göttingen, 37070, Germany; 2 Know-Center GmbH, Graz, 8010, Austria; 3 Copernicus Publications, Göttingen, 37081, Germany; 4 BioMed Central, London, N1 9XW, UK

**Keywords:** open peer review, open science, good practice, research integrity

## Abstract

Open peer review (OPR), as with other elements of open science and open research, is on the rise. It aims to bring greater transparency and participation to formal and informal peer review processes. But what is meant by `open peer review', and what advantages and disadvantages does it have over standard forms of review? How do authors or reviewers approach OPR? And what pitfalls and opportunities should you look out for? Here, we propose ten considerations for OPR, drawing on discussions with authors, reviewers, editors, publishers and librarians, and provide a pragmatic, hands-on introduction to these issues. We cover basic principles and summarise best practices, indicating how to use OPR to achieve best value and mutual benefits for all stakeholders and the wider research community.

## Introduction

Peer review is heralded as the bedrock of quality assurance in scholarly communication, used to scrutinise, select, and improve manuscripts for publication (and further applied in many other contexts, including the review of grant proposals, conference papers, etc). However there are differences in the way various models of peer review are implemented. What is often termed ‘traditional’ or ‘conventional’ peer review is generally (1) partially or completely anonymous, with either the reviewer unknown to the author (single-blind review) or both author and reviewer unknown to each other (double-blind review); (2) selective, with reviewers invited by editors; and (3) opaque, with neither the review, editorial process nor the review reports themselves ever made public. Large-scale surveys continuously show that researchers hold peer review to be beneficial, but that processes are potentially often sub-optimal and open to criticism for being, for example, biased or slow (e.g. 1, 2, 3). In response to these criticisms, and as the wider agenda towards open research is taking hold, variations of open peer review (OPR) are increasingly being offered by publishers and third-party vendors as a regular or additional feature of the publication process.

So what is OPR? OPR means different things to different people and communities and has been defined as “an umbrella term for a number of overlapping ways that peer review models can be adapted in line with the aims of open science” ^4^. Its two main traits are “open identities”, where both authors and reviewers are aware of each other’s identities (i.e., non-blinded), and “open reports”, where review reports are published alongside the relevant article. These traits can be combined, but need not be, and may be complemented by other innovations, such as “open participation”, where the wider community are able to contribute to the review process, “open interaction”, where direct reciprocal discussion between author(s) and reviewers, and/or between reviewers, is allowed and encouraged, and “open pre-review manuscripts”, where manuscripts are made immediately available in advance of any formal peer review procedures (either internally as part of journal workflows or externally via preprint servers).

All these features aim towards either increased transparency, rigour, or inclusivity in research processes, as well as recognizing reviewers’ contribution to published research literature, driven by a wide range of considerations. In this sense, this article sets out the following 10 items that outline how to apply OPR in a way such that it becomes a valuable exercise for you as an author, reviewer or editor.

The results of this papers have been derived from a review of a broad range of research studies (in some the authors have been involved), as well as practices and experiences at publishing houses that have implemented OPR (including those to which the authors are affiliated, for the literature review we build on ^4^).

## Item 1: Understand what kind of peer review you’re dealing with

The term “open peer review” is often used to refer to a number of distinct innovations in peer review, which are combined in many different ways ^4^. As an author, reviewer or editor it is essential that you take the time to understand the choices and obligations you have under each system. Must reviewer identities be revealed or is this optional? Will reviewer reports be published upon acceptance, or even if manuscripts are rejected? Will authors and reviewers be brought into discussion with each other? Familiarising yourself with the particular aspects of an OPR process will help avoid any surprises later on. If in doubt, do not hesitate to contact the journal editor to clarify any questions. Editors play an important role in moderating the review process and are glad to provide additional guidance.

## Item 2: Open peer review relies on, and encourages mutual trust, respect, and openness to criticism

Whatever the degree of openness in a peer review process, as a standard form of academic best practice, it is essential to act with an attitude of charity and in accordance with the highest moral principles ^5^. First of all, as a reviewer you should start with acknowledging the authors’ effort in presenting their results — i.e. review on the assumption that the text makes sense, that it is important and interesting, even if it does not seem so at first glance (cf. Davidson’s “principle of charity” ^6^). In the review process, authors and reviewers typically collaborate on the improvement of a manuscript, which is in principle submitted as ready for publication. A notable exception here is the registered report article format in which the rationale for a study and the proposed methodology — the “study protocol” — are pre-registered with the journal and submitted for peer review before the actual gathering of data and research takes place ^7^. During the review process, authors and reviewers may subjectively agree or disagree on how to present the results and/or what needs improvement, amendment or correction. Now imagine all this happens with readers able to see the process ‘live’ or after the article has been published! It is therefore essential that reviewers ensure that they communicate their points in a clear and civil way, to maximise the chances that it will be received as valuable feedback by the author(s). Authors should also be able to respond in kind (i.e., treating peer review as a dialogue, not a monologue), accepting comments and critique as a process of constructive collaboration in ensuring their work is of the highest quality for publication, and refrain from anything which could be interpreted as a vengeful action.

## Item 3: Open peer review enables constructive and efficient quality assurance

In situations where manuscripts are made available as a preprint and review reports are disclosed, all steps of the scientific quality assurance process can be traced and examined. Experiences gained since the early 2000s have shown that submissions that are posted publicly for interactive public peer review start off with a higher quality compared to those submitted in closed peer review processes ^8^. Although some early studies found no overall change in quality between single-blind versus identities revealed to the authors (i.e. “open identities”) ^9^, other studies have indicated that there may be an improvement of the overall quality of review reports under OPR, particularly that comments are more constructive ^10, 11^.

Some OPR approaches rely on a consensus-building process between the reviewers (e.g., eLife takes this approach), which is an efficient way of providing feedback to the authors. Consistent feedback saves the authors’ time and is far easier to take into account than contradictory reports. Signed reviews enable direct communication between authors and reviewers and thus may also enhance constructive quality assurance, although there is a risk that non-anonymous reviews will be less critical.

OPR can also address aspects of the replication crisis making it easier for experts and non-experts to evaluate the reliability of findings.

## Item 4: Open peer review increases transparency and accountability

Transparency in peer review — i.e. with review reports and/or reviewer identities disclosed — can be beneficial for all parties –authors, reviewers, editors, and readers. Transparency of the peer review process enables readers to see how any (dis)agreements (and potential biases) were addressed and how the final version emerged (who argued for what, what arguments were adopted, how controversial points were addressed, etc.). This is especially the case when the authors’ responses to the reviewers’ comments are also published. An OPR process also makes the reviewers accountable for their comments, and indeed the editor accountable for their choice of reviewers and the final acceptance decision. On this basis, the research community and wider public can assess all comments made by authors, reviewers and editors, and may even participate in the discussion. By fostering such transparency, OPR reports can help dispel persistent concerns about the rigour of peer review processes ^12^, or even highlight places where these concerns might be perfectly legitimate.

In fact, were OPR the standard, it could help distinguish journals, authors, editors, and reviewers who follow good practices from those that do not. Furthermore, the growing body of openly available publications together with review reports also enables the mining of content and perceptions as well as directions of research, including the assessment of “quality and quantity of contributions to the peer-review process alongside publication record as an additional measure of a researcher’s impact in his or her field” ^13^. However, how to best archive and preserve published review reports and related comments has yet to be addressed comprehensively.

## Item 5: Open peer review facilitates wider, and more inclusive discussion

Research findings emerge in a complex network of scholarly communication, but only a small part of this process is currently recorded and made publicly available to authors, reviewers, and readers. Publicly available peer review reports, comments and discussions broaden the perspective on the research presented. They document how reviewers and authors, as representatives of the research community, evaluated the work’s achievements, merits and shortcomings in its early stages of being ‘made ready’ for publication. Depending on the topic, papers may receive from just a few to up to a hundred comments ^8^ (for a paper which received many comments see e.g. 14). Again, journal editors can play an important role in encouraging and moderating comments from the research community and the wider public.

If the process is opened up to the wider community, additional constructive input (in addition to the reviewer reports) can help to further enhance the quality of a manuscript. Examples for journals with such discussions are the Journal Economics, the interactive journals of Copernicus Publications, and SciPost. This type of open commenting facilitates discussion and (in some approaches) consensus building between reviewers and authors. Finally, as an author, you may cite reviews and comments in your revised version, and thus acknowledge these contributions.

## Item 6: Open peer review gives reviewers recognition and makes reviews citable

Peer review, done well, is hard work — usually taking between 5 hours (median) and 8.5 hours (mean) per person per review ^15^. Yet, traditionally there have been few obvious incentives for reviewers, beyond a quid pro quo status of mutualism. A recent survey of almost 3,000 reviewers found that 4 in 5 agreed peer review is currently insufficiently recognised, and that reviewers would invest extra effort if review activities were formally acknowledged in research assessments, promotion processes and funding applications ^16^. OPR can facilitate this by making review activities visible, open to inspection, and formally citable (e.g. by assigned DOIs to review reports). Traditionally, as a reviewer, it was only possible to indicate the journals that one had reviewed for — now with open reports linked to reviewer identities, reviews become creditable research outputs in their own right. Crossref has recently adopted a metadata schema that allows DOIs to be assigned to reviews, enabling peer review reports to be persistent research outputs, which can be listed on CVs and ORCID profiles ^17, 18^.

## Item 7: Open peer review is gaining popularity

While OPR is not without its challenges (e.g. 19, 20, 21), support is growing across various fields. It has long been recognized to be feasible in practice ^13^ and in recent optional trials, the majority of authors are willing to publish the full peer review history if given the opportunity (e.g. 22, 23). However, there are still strong concerns against disclosing reviewer identities, with more than half believing it would make peer review worse ^24^. Anecdotal evidence from editors suggests it can be harder to find reviewers who are willing to agree to OPR, however this is not insurmountable in practice ^25^ and can be outweighed by the advantages discussed above. OPR is certainly happening across many research fields and looks set to rise in the future ^26^.

## Thing 8: Open peer review offers learning opportunities and facilitates training

With every review process, both early career and established reviewers can learn something, typically about a new finding in their field of research but also through providing feedback and advice to other researchers. Where reports of fellow reviewers are made available, it is worthwhile to read them carefully and learn from them in writing your next review ^5^. OPR opens up this opportunity to a wider audience, and adds further learning points. Consider using open review reports as instructive material in training sessions with early career researchers on how to write a good review. Open review comments offer further opportunities: they can serve as a testing ground for brief feedback or more comprehensive additional reviews.

However, as an early career researcher you may not feel comfortable to step into a fully open review process, in particular if you have strong opinions about the paper under review. Perhaps, a potential compromise is to start with a journal that allows you to become gradually open. For example, Royal Society Open Science and PeerJ encourage but do not require reviewers to sign their reports, and authors are allowed to decide whether reviewer reports and author responses are published alongside articles. In addition, turning to a trusted mentor for advice whilst undertaking a first-time OPR may be helpful.

## Item 9: There is room to practice open peer review even if it has not been formally introduced yet

Even where journals do not formally operate an OPR process, as an author or reviewer you will still have room to practice openness. For example, you can often choose to sign your review, although this can depend on the individual editor or peer review workflow whether this information will be passed to the author. When signing reviews you may consider doing so with reference to the Open Science Peer Review Oath ^27^. A more radical step is for a reviewer to publish the review report, however, this would require express agreement in advance with the authors and journal.

The most radical approach from a reviewer perspective was recently featured in WIRED magazine: neuroscientist Niko Kriegeskorte advocates only reviewing manuscripts that have already been made available online on a preprint server. When he receives a review request, he first emails the author to identify himself and advise of his review policy, requesting the author upload a preprint online. He then reviews the paper, posts the review on his blog and in conclusion sends the review to the editor ^28^. In a similar spirit, the PRO initiative encourages reviewers to make openness of underlying research data and related materials a pre-condition of comprehensively reviewing a paper.

As an author there is less room for direct interaction in the traditional journal setting but with the emergence of preprint servers in many fields you have new opportunities to collect feedback and comments from the scientific community and wider public, both in public and private. If you want to crowd-source additional comments and reviews for your manuscript via open participation, this is still possible even if the venue you submit to does not practice such peer review — simply post your pre-review manuscript to a preprint-server simultaneously with submission and invite feedback through commenting features, e.g. services for collaborative review of preprints ^29^, or just ask people to email or contact you via social media (again, do acknowledge these contributions in your revised version). However, as some journals consider posting on a preprint server as pre-publication you need to check beforehand if your preferred journal permits posting on preprint servers (cf. the SHERPA RoMEO database and Wikipedia’s Academic journals by posting policy).

## Item 10: We need more analysis of and research on open peer review

It is well recognized that the diverse practice of peer review is not without its flaws (for a summary see e.g. 30). Although OPR may help address some of them, it will not solve them all or suit every community. OPR can provide incentives for robust open research practices but will not be able to completely prevent undesirable behavior or even misconduct. However, it provides a means to make such cases much more transparent, even in cases of retractions (for an example discussed on Retraction Watch see 31).

There is still need for further research into OPR, especially in terms of both desirable and unintended outcomes as well as efficacy compared to conventional processes. We need additional evidence from authors, reviewers and editors on how effective OPR actually is in various fields. We also need further research into which issues and biases in peer review still need to be addressed ^4, 24, 32^, and how the publication environment can be further improved in order to better support diversity and inclusivity in peer review. OPR is not a panacea and other models, experiments and initiatives with peer review may help to address some of the concerns with traditional peer review.

## Discussion

In this section we would like to briefly outline where further investigation is needed especially with regard to unintended effects, possible biases, how to mitigate those effects and what role “opt-out” may play in the above good-practice recommendations.

Are there any rules missing? Possible “Opt out if there is a sound reason to do so” could be considered. For example, in pilots with OPR experiments ^10^, about 1 in 10 reviewers declined to review due to a potential conflict of interest and 1 in 4 for personal reasons. However, time was the most important factor (over 2/3). In the context of this paper, we have refrained from introducing an “opt out” related to any of the traits of OPR as the option to decline to review seems sufficient.

One further open question is who benefits most from OPR, e.g. is it the case that there are more positive reviews for well-established researchers or for papers which tackle more trendy topics? Which biases play out in OPR? Are language skills a factor? Are there country, subject or gender biases? How can such effects be controlled/mitigated? For the latter there certainly is a role for e.g. editors, who can amend policies and provide additional instruction and education. Is “open reports” the best we can have given the strong competition in academia?

Some of these questions are currently being explored, e.g. in the context of the EU-funded OpenUp project. In addition to exploring workflows and the behavior of all actors involved, OpenUp proposes to collect and aggregate data across publishers in order to evaluate the efficacy of OPR processes ^33^.

However, the good news is that in OPR settings biases can be monitored and inform interventions. E.g. a recent study revealed that all-female economics papers remain six months longer in peer review than all-male papers, and the author expressed that hope that open peer review may prevent such behavior ^34^. An investigation based on Wellcome Open Research from the first year of operation (based on 142 papers, gender of first author) showed that reviewers took only a few days longer to review papers of female first authors (19.5 vs. 14 days for female vs. male first authors) ^35^. Further investigation would be desirable, in order to monitor undesirable behavior and identify opportunities for editor intervention.

## Conclusion

OPR is an innovation in scholarly communication that deserves further attention. As we have outlined in the 10 items above, it places a research work in the context of a discussion, and gives authors, readers and others a chance to better understand the process from the initial manuscript submission to final published version. As such it provides excellent learning opportunities and the potential to improve scholarly communication and research towards a more transparent, collaborative and participative undertaking.

## Data availability

No data are associated with this article.
