# Supplementary material for: Ten considerations for open peer review
Source: F1000Res. 2018 Jun 29;7:969. [Version 1] doi: 10.12688/f1000research.15334.1 (PMC6073088; doi:10.12688/f1000research.15334.1)
Supplement: Peer review history

# Peer review: Referee response for version 1

**DOI:** 10.5256/f1000research.16707.r35998

**Published:** 2018-08-01

**Authors:** Darko Hren

**Affiliations:** 1 Faculty of Humanities and Social Sciences, University of Split, Split, Croatia

This is an nice introductory article to a reader who wishes to learn about open peer review. Authors introduce the main concepts and lay down ten items to be considered when dealing with open peer review. It will be useful to early stage researchers and  general readership new to the issue.

 This is not a research article, but a non-systematic review of existing literature with recommendations from experienced researchers and professionals in the field.

 In my opinion, the article can be indexed without revisions.

 Minor point:

-  pp 4 bottom right says "Thing 8" - should be "Item 8" (?)

I have read this submission. I believe that I have an appropriate level of expertise to confirm that it is of an acceptable scientific standard.

# Peer review: Referee response for version 1

**DOI:** 10.5256/f1000research.16707.r35996

**Published:** 2018-07-23

**Authors:** Richard Smith

**Affiliations:** 1 International Centre for Diarrhoeal Disease Research, Dhaka, Bangladesh

I'm glad that this article is already available to be read by those who want to know more about open peer review, particularly young researchers. The article is a useful guide.

 Ironically, I think that little value will be added by me peer reviewing it openly ot otherwise. If the article had been submitted to a more traditional journal, including many that review openly, then it would not be available for people to read. It might have languished for months while it progressed slowly through the bureaucracy of science journals. Indeed, it would probably be rejected because there is little or nothing that is new in the article. The article would then work its way down the food chain of journals before appearing in an obscure journal where few people would read it, especially if it appeared in a journal behind a paywall.

 Why have the authors submitted the paper to a journal? Why haven't they simply posted it as a blog and used social media to promote the article? They might easily achieve a higher readership, and presumably it is readers that they want. Or is it? I think that these authors are primarily interested in readers, but sadly the main motivation for many authors is simply to add to their CVs and increase their chances of raising more grants and getting promoted.

 I am letting my cynicism about the whole process of science publishing shine through, and I would have liked this article more if it had been less accepting of the status quo and more questioning. But that is probably not the role of a simple guide.

 I don't have any major criticisms of the article, and it will be fine if it published and indexed as it is. But here are some comments in descending order of importance.

 1. I think that the authors could have done a better job of discerning the quality of the evidence they quote. As I've argued elsewhere peer review is more faith than evidence based, https://breast-cancer-research.biomedcentral.com/articles/10.1186/bcr2742 and scientists who would not advance string views on their own subject without evidence are quite happy to make statements on peer review without  evidence. For example, in Item 3 the authors write: “Although some early studies found no overall change in quality between single-blind versus identities revealed to the authors (i.e. “open identities”), other studies have indicated that there may be an improvement of the overall quality of review reports under OPR, particularly that comments are more constructive.” They do not make clear that the first study was a randomised trial, a much more reliable method than the later studies, one of which was simply a survey and the other a retrospective study.

 2. This is not a scientific study, but the authors do outline what might be  described as their methods in the fourth paragraph. They might usefully expand this, providing more information and explaining how this paper builds on their systematic review.

 3. This is my bias, but I'd have liked to see a short section pointing out that there is little empirical as opposed to anecdotal evidence to support peer review but lots demonstrating its problems. At the end the cite my article in the JRSM, but they would do better to cite my article in Breast Cancer Research ^1^, which is more up to date and contains more data. They might also have discussed more the objectives of peer review.

 4. The authors suggest that tyro reviewers might start with journals, like *Royal Society Open Science* that allows reviewers to choose whether or not to be named. This is a tangential point, but I think that offering this option is probably a bad idea. I've experienced a case where a journal rejected a paper on the basis of an unsigned review while also sending the authors a glowing signed review. You can imagine that this seemed unfair to authors. The other strategy the authors suggest--of consulting a mentor--is a much better idea.

 5. Perhaps the authors might say more about the ethics of open peer review. We introduced open peer review at the BMJ when I was the editor largely on ethical grounds as our studies did not show that open peer review was superior to closed peer review ^2^. All publishing should be about credit and accountability, and closed review is weak on both.

 6. I, like most readers, find it annoying when authors introduce things I've never heard of without saying more. I'd like them not just to give a reference to Open Science Peer Review Oath but tell me a bit more in this paper. Similarly they refer to "the PRO initiative." Is PRO  a reference to the Peer Review Oath? I guess that it probably is, but I don't want to have to guess.

 7. I wish the authors had not succumbed to the academy disease of acronyms. I'd lime them to spell out OPR, and there is something particularly silly about referring, as they do, to "the process of OPR."

 8. I hadn't heard of the process the authors mention adopted by Niko Kreigeskorte, but I think it a great idea. Like him I will post these comments on my blog as well as on *F1000Research*.

 Conclusion

 As I said at the beginning, I’m sure that some researchers and others will find this guide useful, and I’m glad that it’s already published. The authors might want to revise the paper in the light of my comments, but I don’t think that it’s essential. I support the paper being indexed whether or not they revise the paper.

I have read this submission. I believe that I have an appropriate level of expertise to confirm that it is of an acceptable scientific standard.

## References

[1] SmithR: Classical peer review: an empty gun. *Breast Cancer Res*.2010;12 Suppl 4: 10.1186/bcr2742 S13 10.1186/bcr2742 21172075PMC3005733

[2] SmithR: Opening up BMJ peer review. *BMJ*.1999;318(7175) : 10.1136/bmj.318.7175.4 4-5 10.1136/bmj.318.7175.4 PMC11145359872861
